# PRMT5 regulates cell pyroptosis by silencing CASP1 in multiple myeloma

**DOI:** 10.1038/s41419-021-04125-5

**Published:** 2021-09-16

**Authors:** Tian Xia, Ming Liu, Quan Zhao, Jian Ouyang, Peipei Xu, Bing Chen

**Affiliations:** 1 Department of Hematology, The Affiliated Drum Tower Hospital of Nanjing University Medical School, Nanjing, 210008 Jiangsu People’s Republic of China; 2 The State Key Laboratory of Pharmaceutical Biotechnology, Nanjing University, Nanjing, 210008 People’s Republic of China; 3 Clinical College of Traditional Chinese and Western Medicine, Nanjing University of Chinese Medicine, Nanjing, 210008 Jiangsu People’s Republic of China

**Keywords:** Myeloma, Enzyme mechanisms

## Abstract

Protein arginine methyltransferase 5 (PRMT5), a histone methyltransferase responsible for the symmetric dimethylation of histone H4 on Arg 3 (H4R3me2s), is an enzyme that participates in tumor cell progression in a variety of hematological malignancies. However, the biological functions of PRMT5 in multiple myeloma (MM) and the underlying molecular mechanisms remain unclear. In this study, we conducted a bioinformatics analysis and found that PRMT5 expression was significantly upregulated in MM. In vitro and in vivo phenotypic experiments revealed that knockdown of PRMT5 expression enhanced cell pyroptosis in MM. Moreover, we found that CASP1 expression was negatively correlated with PRMT5 expression, and repressing PRMT5 expression rescued both the phenotype and expression markers (N-GSDMD, IL-1b, and IL-18). Inhibition of PRMT5 activity increased CASP1 expression and promoted MM cell pyroptosis. Finally, high expression of PRMT5 or low expression of CASP1 was correlated with poor overall survival in MM. Collectively, our results provide a mechanism by which PRMT5 regulates cell pyroptosis by silencing CASP1 in MM.

## Introduction

Multiple myeloma (MM) is a malignancy that originates from the extensive proliferation of pathological antibody-producing plasma cells in the bone marrow and accounts for ~10% of hematological cancers [1]. From 2006 to 2016, 16,500 new cases of MM were reported in China, and the age-standardized mortality rate (ASMR) was 0.67/100,000 in 2016 [2]. Many patients with MM suffer from bone destruction and hypercalcemia as a result of osteoclast activation and osteoblast inhibition caused by cytokines expressed by MM cells [3], and from renal failure as a result of the accumulation and precipitation of light chains produced by high amounts of MM cells [4]. At present, many studies have proven that individuals with MM share the same genetic features; thus, MM is thought to arise as a result of the accumulation of genetic alterations. Hypermethylation of many tumor suppressor genes, such as SOCS-1 [5, 6] and RUNX2 [7], has been shown to contribute to MM progression [8]. Nevertheless, the development of MM is a complicated pathological process that requires further study.

Protein arginine methyltransferase 5 (PRMT5) is the main type II methyltransferase that catalyzes the symmetric addition of dimethylarginine to histone proteins, nonhistone proteins, and cytoplasmic proteins and is involved in numerous cellular processes, including transcription, DNA repair, RNA processing, proliferation, and metabolism [9–11]. Several studies have demonstrated that PRMT5 is overexpressed in hematological malignancies, including leukemia, lymphoma, and MM [12]. Gullà et al. [13] have found that the PRMT5 inhibitor EPZ015666 induces cell autophagy via the PRMT5/TRIM21/IKKB axis, which is identifying PRMT5 as a promising therapeutic target in MM. However, there is still a lack of detailed evidence to explain how PRMT5 triggers the proliferation of MM cells in detail.

In this study, in addition to our cohort, we extracted clinical and RNA sequencing data from the Multiple Myeloma Research Foundation (MMRF) CoMMpass study to assess the clinical significance of PRMT5. We also explored the biological function of PRMT5 in MM cells and investigated the mechanism by which PRMT5 regulates MM cell pyroptosis by repressing CASP1.

## Results

### PRMT5 was overexpressed in MM

**Fig. 1:**
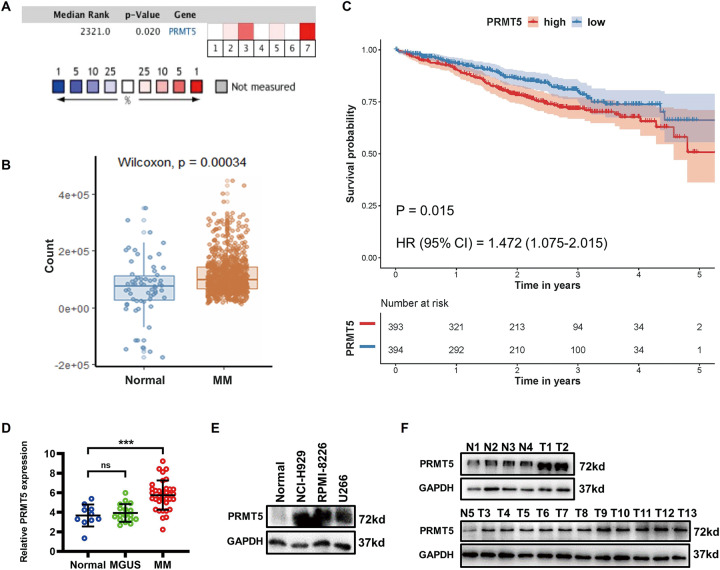
PRMT5 is overexpressed in MM. **A** PRMT5 expression in 7 MM files from the Oncomine. **B** Relative expression of PRMT5 in MM tissues (*n* = 859) and noncancerous tissues (*n* = 62) from the MMRF CoMMpass and GTEx databases. **C** Progression-free survival times in MM patients with low versus high expression of PRMT5 assessed by Kaplan-Meier analysis from the MMRF CoMMpass cohorts. **D** Relative mRNA expression of PRMT5 in MM (*n* = 35) and MGUS (*n* = 17) tissues compared with that in noncancerous (*n* = 10) tissues via qRT-PCR. **E** PRMT5 protein level in 13 MM tissues and 5 normal tissues via western blotting. **F** PRMT5 protein level in normal plasma cells and 3 MM cell lines (NCI-H929, RPMI-8226, and U266) via western blotting. Data shown are mean ± SD. ***P < 0.001.

A bioinformatics analysis was performed on seven MM datasets from the Oncomine database. Consequently, PRMT5 was found to be significantly upregulated in MM tissues compared to noncancerous tissues (*P* = 0.020, Fig. 1A). In addition, further bioinformatics analysis combining the MMRF CoMMpass (*n* = 859) and Genotype-Tissue Expression (GTEx) databases (*n* = 62) was conducted, which revealed that PRMT5 expression was higher in MM tissues than in noncancerous tissues (*P* = 0.00034, Fig. 1B). Moreover, by analyzing the clinical data from 787 patients with MM from the MMRF CoMMpass database, we found that high PRMT5 expression was associated with poor progression-free survival (*P* = 0.015, HR = 1.472, Fig. 1C). We determined the expression of PRMT5 mRNA in bone marrow-derived plasma cells from 35 MM samples, 17 MGUS samples, and 10 noncancerous samples using quantitative real-time polymerase chain reaction (qRT-PCR) analysis to verify the database results. PRMT5 levels were significantly higher in MM samples than in control samples, while no significant change was observed in MGUS samples (Fig. 1D). Also, datasets including MGUS samples (GSE6477 and GSE5900) showed no difference in PRMT5 expression between healthy donors and MGUS patients. In addition, by using western blotting assays, the PRMT5 protein was found to be overexpressed in MM samples and MM cell lines (Fig. 1E, F).

### Knockdown of PRMT5 triggered cell membrane rupture and elevated PI+/Annexin V+ population in MM cells

**Fig. 2:**
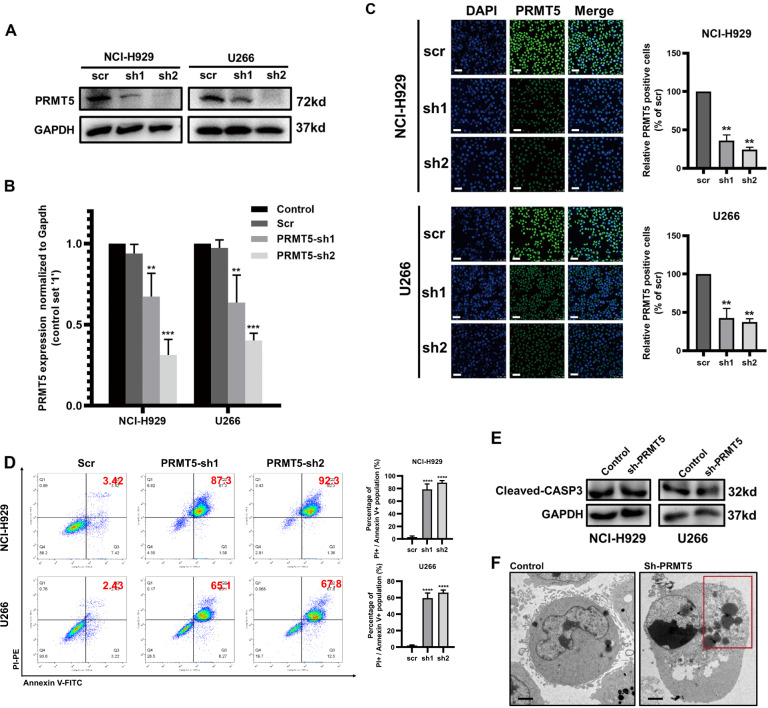
PRMT5 activates pyroptosis of MM cell lines. **A**–**C** PRMT5 knockdown was detected in NCI-H929 and U266 cells stably expressing the scrambled control (scr) or PRMT5 shRNA (PRMT5 sh1/2) by western blotting, qRT-PCR, and immunofluorescence assay. Scale bar = 25 μm. **D** The analysis of Annexin V-FITC and PI by flow cytometry in NCI-H929 (upper panel) and U266 (lower panel) cells stably expressing the control or PRMT5 shRNA. **E** Expression level of cleaved-CASP3 in shPRMT5 cells and negative control cells via western blotting. **F** Representative images of electron microscopy analysis of pyroptosis morphology changes in NCI-H929 cells. Scale bar = 2 μm. Data shown are mean ± SD (*n* = 3). **P < 0.01, ***P < 0.001, ****P < 0.0001.

When investigating the function of PRMT5 in MM cells, we designed two specific short hairpin RNAs (shRNAs) and successfully knocked down the relative levels of PRMT5 expression in two MM cell lines (NCI-H929 and U266) compared to that in the negative control group as assessed by western blotting and qRT-PCR (Fig. 2A, B).These results were further confirmed by immunofluorescence-targeting PRMT5 in both cell lines (Fig. 2C).

Cell Counting Kit-8 (CCK-8) assays indicated that downregulation of PRMT5 promoted cell viability in the two individual MM cell lines (*P* < 0.05, Supplemental Fig. 1A). In addition, knockdown of PRMT5 was confirmed to increase the PI+ population of NCI-H929 and U266 cells by flow cytometry, particularly in the PI+/Annexin V+ domain (Fig. 2D). This phenotype could refer to late apoptosis or pyroptosis, both of which manifest as the coloration of PI dye caused by the poles of cell membrane. Since the activation of caspase 3 (CASP3) is a trigger of the execution pathway in apoptosis, we analyzed the expression of cleaved-CASP3 in shPRMT5 cells and negative control cells, but the results showed no significance (Fig. 2E). Furthermore, TEM images revealed shPRMT5 cells with cytoplasmic swelling and plasma membrane rupture, which supported the morphology of pyroptosis (Fig. 2F). These results suggested that knockdown of PRMT5 activates pyroptosis in MM cell lines.

### PRMT5 upregulation was inversely correlated with CASP1

**Fig. 3:**
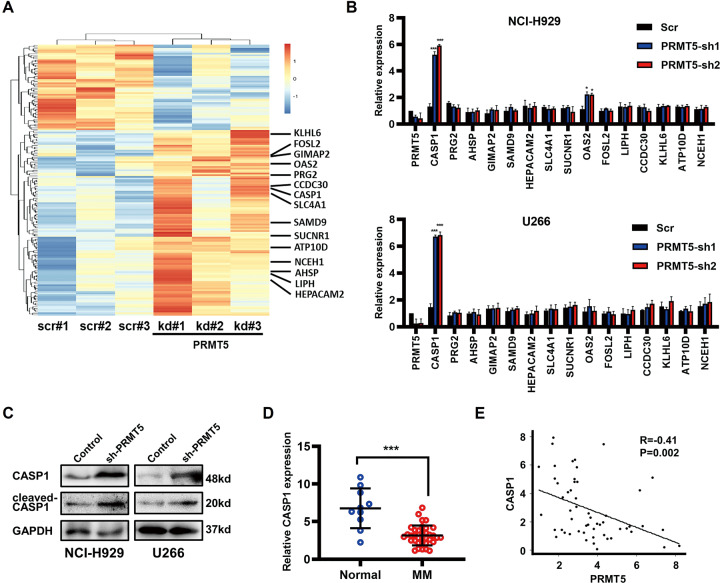
PRMT5 upregulation is inversely correlated with CASP1. **A** Heatmap showing differentially expressed genes after PRMT5 knockdown. Highlighted genes are the top 15 significantly upregulated genes in NCI-H929 cells stably expressing PRMT5 shRNA. **B** Relative mRNA expression levels of significantly upregulated genes in NCI-H929 cells or U266 cells stably expressing PRMT5 shRNA compared to scr shRNA via qRT-PCR. GAPDH was used as an endogenous control. **C** Expression levels of CASP1 and cleaved-CASP1 were both upregulated in NCI-H929 cells or U266 cells stably expressing PRMT5 shRNA than scr shRNA via western blotting. **D** Relative mRNA expression of CASP1 was downregulated in MM (*n* = 35) tissues compared with that in noncancerous (*n* = 10) tissues by qRT-PCR. **E** The Pearson’s correlation analysis of CASP1 and PRMT5 in bone marrow tissues of MM patients (*n* = 55) from the MMRF CoMMpass database. Data shown are mean ± SD (*n* = 3). *P < 0.05, ***P < 0.001.

We conducted a genome array on three NCI-H929 cell samples and three PRMT5-knockdown (PRMT5-kd) NCI-H929 cell samples using Affymetrix Clariom S array chips to determine the function of PRMT5 in MM pathogenesis (Fig. 3A). Datasets and profiles were uploaded to the Gene Expression Omnibus database (GSE162715). Relative gene expression changes of the top 15 significantly upregulated genes in the PRMT5-kd group were explored, including CASP1, PRG2, AHSP, GIMAP2, SAMD9, HEPACAM2, SLC4A1, SUCNR1, OAS2, FOSL2, LIPH, CCDC30, KLHL6, ATP10D, and NCEH1. Results revealed that CASP1 was significantly increased in both NCI-H929 and U266 cells (Fig. 3B). To confirm that CASP1 acts as a downstream target of PRMT5, we evaluated protein expression levels of CASP1 and cleaved-CASP1 in MM cell lines after transfection with PRMT5-shRNA-2 compared to the negative control groups. Notably, knockdown of PRMT5 led to significant elevation of CASP1 and cleaved-CASP1, indicating that CASP1 was silenced by PRMT5 in MM cells (Fig. 3C). In addition, CASP1 mRNA expression in bone marrow-derived plasma cells was significantly decreased and negatively correlated with PRMT5 in patients with MM (Fig. 3D, E).

### Pyroptosis induced by depletion of PRMT5 was rescued by knockdown of CASP1 in MM cell lines

**Fig. 4:**
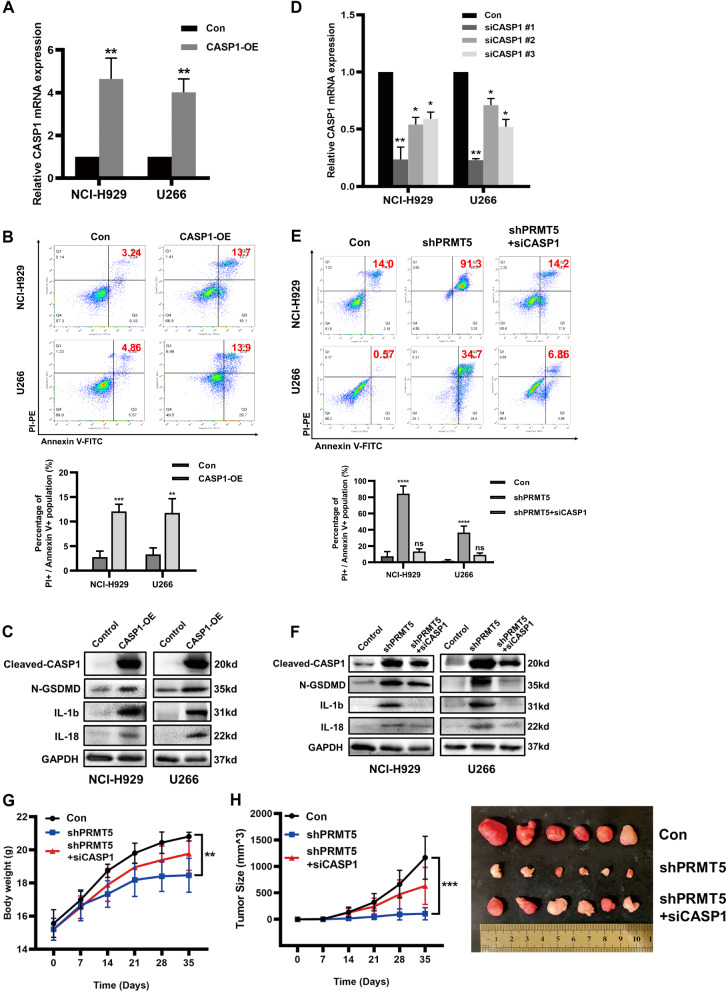
Cell pyroptosis induced by depletion of PRMT5 was rescued by knockdown of CASP1. **A** Relative mRNA expression of CASP1 in NCI-H929 cells and U266 cells transfected with the control plasmid (Con) or CASP1 overexpression plasmid (CASP1-OE). **B** The analysis of Annexin V-FITC and PI by flow cytometry in NCI-H929 (upper panel) and U266 (lower panel) cells transfected with the control or CASP1-OE plasmid. **C** Western blotting detection of cleaved-CASP1, N-GSDMD, IL-1b, and IL-18 in NCI-H929 and U266 cells transfected with the control or CASP1-OE plasmid. **D** Relative mRNA expression of CASP1 in NCI-H929 cells and U266 cells transfected with the control plasmid (Con) or CASP1 siRNAs (siCASP1 #1/#2/#3). **E** The analysis of Annexin V-FITC and PI by flow cytometry in NCI-H929 (upper panel) and U266 (lower panel) cells transfected with PRMT5 shRNA with or without the co-transfected CASP1 siRNAs. **F** Western blotting detection of Cleaved-CASP1, N-GSDMD, IL-1b, and IL-18 in NCI-H929 and U266 cells transfected with PRMT5 shRNA with or without the co-transfected CASP1 siRNAs. **G** Weight curves of xenograft mice carrying scr, shPRMT5, or shPRMT5 + siCASP1-transfected NCI-H929 cells. **H** Images and growth curves of xenograft tumors treated with scr, shPRMT5, or shPRMT5 + siCASP1-transfected NCI-H929 cells. Data shown are mean ± SD (*n* = 3). *P < 0.05, **P < 0.01, ***P < 0.001.

To investigate whether PRMT5 regulates cell pyroptosis by repressing CASP1 expression, we transiently overexpressed CASP1 in NCI-H929 and U266 cells (Fig. 4A). Our results revealed that CASP1 overexpression significantly suppressed cell viability (Supplemental Fig. 1B) and increased the population of PI+/Annexin V+ cells in NCI-H929 and U266 cells (Fig. 4B). Our results also showed that overexpression of CASP1 enhanced the activation of GSDMD and the secretion of interleukin-1 beta (IL-1b) and interleukin-18 (IL-18) (Fig. 4C). Similarly, CASP1 expression was elevated in PRMT5-depleted cells, which activated pyroptosis pathways. However, when CASP1 was knocked down by siRNAs in PRMT5-depleted cells (Fig. 4D), markers of the pyroptosis pathway were significantly downregulated, and the percentage of pyroptotic cells was remarkably decreased (Fig. 4E, F). Cell growth was rescued after depletion of CASP1 in shPRMT5 cells (Supplemental Fig. 1C). Furthermore, we performed in vivo experiments using mouse xenograft models and found that the knockdown of CASP1 partially reversed the proliferation defect in PRMT5-depleted NCI-H929 cells in vivo (Fig. 4G, H). The results described above demonstrated that cell pyroptosis induced by depletion of PRMT5 is rescued by knockdown of CASP1.

### Treatment with the PRMT5 inhibitor GSK591 reduced the level of H4R3me2s at the CASP1 promoter

**Fig. 5:**
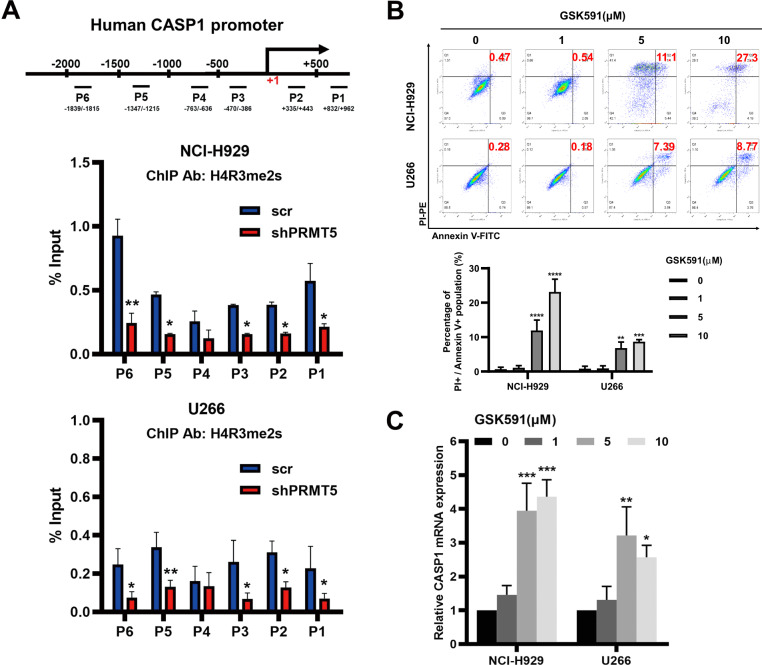
Treatment of PRMT5 inhibitor GSK591 reduces the level of H4R3me2s at the CASP1 promoter. **A** Schematic drawing of the CASP1 promoter region. The positions of ChIP-qPCR primer set were labeled relative to the transcriptional start site (TSS). TSS is assigned as the ‘+1’ position. PRMT5-mediated H4R3me2s modifications were significantly less enriched at the core promoter regions of CASP1 genes in NCI-H929 cells (upper panel) and U266 cells (lower panel) stably expressing PRMT5 shRNA than scr shRNA by ChIP analysis. IgG was used as a negative control. The bar graph shows percentages of relative fold enrichment of H4R3me2s at different positions across the CASP1 promoter region, compared with the input. **B** The analysis of Annexin V-FITC and PI by flow cytometry in NCI-H929 (upper panel) and U266 (lower panel) cells treated with 0, 1, 5, 10 μM of GSK591. **C** Relative mRNA expression of CASP1 in NCI-H929 and U266 cells treated with 0, 1, 5, 10 μM of GSK591. Data shown are mean ± SD (*n* = 3). *P < 0.05, **P < 0.01, ***P < 0.001.

We designed six pairs of walking primers across the core promoter regions of the CASP1 gene to test whether PRMT5 directly regulates CASP1 expression and analyzed the enrichment of PRMT5-mediated H4R3me2s around the CASP1 gene promoter using chromatin immunoprecipitation (ChIP) assays and qRT-PCR. Our results demonstrated that, in PRMT5-depleted NCI-H929 and U266 cells, the levels of H4R3me2s were significantly reduced at the promoter region of CASP1 compared to the control group (Fig. 5A).

To inhibit H4R3me2s at the CASP1 gene promoter, we treated NCI-H929 and U266 cells with GSK591 (also known as EPZ015866), which is widely acknowledged as a specific inhibitor of PRMT5 [14, 15]. We treated NCI-H929 and U266 cells with 0, 1, 5 and 10 μM GSK591 (B6182, Apexbio) to investigate its biological function. We found that 5 μM GSK591 treatment significantly triggered an increase in the proportion of PI+ cells in both NCI-H929 and U266 cells, suggesting membrane rupture and secondary cell death caused by pyroptosis (Fig. 5B). Next, we examined the expression of CASP1 in NCI-H929 and U266 cells treated with various concentrations of GSK591 to explore whether inhibiting the enzymatic activity of PRMT5 could reactivate CASP1 expression. We found that 5 μM GSK591 increased CASP1 expression (Fig. 5C). Our results indicated that GSK591 reduced the levels of H4R3me2s and accordingly activated CASP1-mediated cell pyroptosis by inhibiting PRMT5.

### High expression of PRMT5 and low expression of CASP1 were associated with poor clinical outcomes in patients with MM

**Fig. 6:**
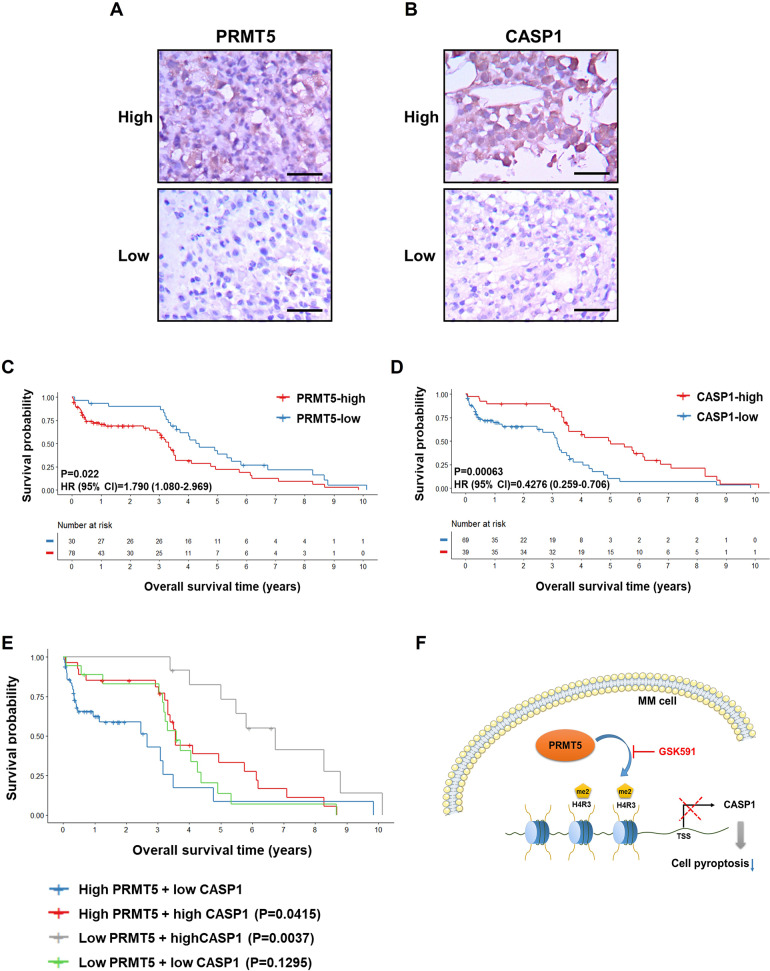
High expression of PRMT5 and low expression of CASP1 associate with poor clinical outcomes in MM patients. **A**, **B** Representative images of IHC staining of PRMT5 and CASP1 in MM bone marrow tissues, which were divided into a high-expression group (upper panel) and a low-expression group (lower panel). Scale bar = 50 μm. **C** Overall survival of high PRMT5 (*n* = 78) and low PRMT5 (*n* = 30) MM patients performed by Kaplan-Meier survival analysis. **D** Overall survival of high CASP1 (*n* = 39) and low CASP1 (*n* = 69) MM patients performed by Kaplan-Meier survival analysis. **E** Kaplan-Meier survival analysis of MM patients based on both PRMT5 and CASP1 expression. MM patients were divided into four groups: high PRMT5 and low CASP1 expression (*n* = 51), high PRMT5 and high CASP1 expression (*n* = 27), low PRMT5 and high CASP1 expression (*n* = 12), and low PRMT5 and low CASP1 expression (*n* = 18). *P*-values were calculated using the group of high PRMT5 and low CASP1 expression as the reference group. **F** A model for PRMT5-dependent cell proliferation and apoptosis by silencing CASP1 in MM.

We collected clinical data from 108 newly diagnosed MM patients in our hospital from 2008 to 2020, and determined the PRMT5 and CASP1 expression levels by immunohistochemistry. Bone marrow tissues from MM patients were divided into PRMT5-high and PRMT5-low groups (Fig. 6A), as well as CASP1-high and CASP1-low groups (Fig. 6B). A significant association between PRMT5 expression and ISS stage was observed in patients with MM (Table 1). Additionally, Kaplan-Meier curves (Fig. 6C, D) demonstrated that high PRMT5 expression (*P* = 0.022) and low CASP1 expression (*P* = 0.00063) were associated with poor overall survival in MM. We merged these two variants and found that patients with both low PRMT5 and high CASP1 expression exhibited significantly worse survival compared with to those carrying high PRMT5 and low CASP1 expression (Fig. 6E, *P* = 0.0037). Moreover, both the univariate (*P* < 0.001) and multivariate (*P* = 0.007) Cox proportional hazards regression analyses demonstrated that PRMT5 upregulation was an independent prognostic risk factor for poor survival in patients with MM (Table 2).

**Table 1:** The relationship between PRMT5 expression and clinicopathological characteristics in MM.

Characteristics	No.	Expression of PRMT5 level in MM		*χ*2	*P*-value
		Low	High		
*N*	108	30	78		
Gender					
Male	65	18	47	0.001	0.981
Female	43	12	31		
Age (years)					
≤60	59	13	46	2.139	0.144
>60	49	17	32		
ISS stage					
I	18	8	10	8.502	**0.014**
II	40	13	27		
III	50	7	43		
M protein type					
IgG type	50	11	39	5.81	0.325
IgA type	30	9	21		
IgM type	1	0	1		
IgD type	5	3	2		
Light chain type	19	7	12		
Non-secretary type	3	0	3		

*MM* multiple myeloma, *ISS* international staging system. Bold values are statistically significant (*P* < 0.05).

**Table 2:** Univariate and multivariate analyses of clinicopathological characteristics for survival in patients with MM.

Characteristics	Univariate analysis *P*-value	Multivariate analysis	
		*P*-value	HR (95% CI)
Expression of PRMT5	**<0.001**	**0.007**	3.940 (1.454–10.678)
Gender	0.424	0.329	1.404 (0.711–2.771)
Age	0.180	**0.008**	0.393 (0.197–0.768)
ISS stage	**<0.001**	**<0.001**	5.091 (2.600–9.969)
M protein type	0.699	0.701	0.958 (0.768–1.194)

*HR* hazard ratio, *CI* confidence interval. Bold values are statistically significant (*P* < 0.05).

## Discussion

In the present study, we observed that PRMT5 expression was significantly upregulated in MM bone marrow tissue, which might serve as an adverse prognostic factor for overall survival in patients with MM. Moreover, PRMT5 suppressed cell pyroptosis pathways in vitro, and enhanced tumor progression in vivo. However, these biological phenotypes were inhibited by the knockdown of PRMT5 and then rescued by overexpressing CASP1 in PRMT5-depleted cells, indicating that PRMT5 regulates cell pyroptosis by silencing CASP1 in MM. This study reported the mechanism and significance of the interaction between CASP1 and PRMT5 for the first time and briefly introduced GSK591 treatment as a potential therapeutic strategy for MM.

Arginine methylation plays a crucial role in posttranscriptional modification [16]. PRMT5, which is the predominant type II methyltransferase-targeting arginine methylation, is upregulated in a variety of hematological malignancies and its overexpression is associated with tumor aggressiveness and poor overall survival [17–21]. Several therapeutic agents targeting PRMT5 have entered early clinical trials [22]. A study by Ludivine et al. [23] revealed that PRMT5 prevents premature plasma cell differentiation. Gullà et al. [13] found that inhibition of PRMT5 by EPZ015666 inhibits MM cell proliferation and decreases tumor growth through TRIM21-dependent NF-κB inhibition. PRMT5 inhibition was also reported to reduce the expression of E2F targets and alter the methylation status of E2F1 in JAK2V617F-mutant myeloproliferative neoplasms [22]. Several studies have supported the ability of PRMT5 to activate the transcriptional regulation of downstream molecules. A study by Ge et al. [24] revealed that PRMT5 induces H4R3 symmetric dimethylation to increase the transcription of FGFR-3 and eIF4E. Liu et al. [11] demonstrated that PRMT5-mediated H4R3me2s epigenetically repressed transcription of the c-Myc target genes, PTEN, p18, p21, p57, and p63, to promote cell proliferation and gastric cancer progression. Deng et al. [25] revealed that PRMT5 interacts with BRG1 and Sp1 to induce symmetrical dimethylation modification of H4R3 in the promoter region of the androgen receptor gene, activating androgen receptors and promoting the proliferation of prostate cancer cells. In our study, we explored the CASP1-mediated pyroptosis pathway and demonstrated that PRMT5-induced H4R3me2s silences the transcription of CASP1 to suppress cellular pyroptosis in MM (Fig. 6F).

PRMT5 induces gene silencing not only by generating repressive histone marks such as H4R3me2s, but also by methylating nonhistone proteins, such as the transcription factors p53 [26, 27], E2F1 [22, 28], and p65 [12, 26], as well as intercellular protein molecules, such as Sm protein [29], ribosomal protein S10 (RPS10) [30], and rapidly accelerated fibrosarcoma (RAF) [31]. Investigating these interactions of genes with PRMT5 in MM will be of great interest.

The CASP1 gene encodes caspase 1, a member of the cysteine-aspartic acid protease (caspase) family which primarily involves the inflammatory process [32]. However, CASP1-mediated cell pyroptosis has also been shown to participate in various tumor development stages [33–35]. CASP1 initiates the pyroptosis pathway through cleavage of GSDMD, and it also proteolytically cleaves the precursors of the inflammatory cytokines IL-1b and IL-18 [36–38] and is essential to distinguish pyroptosis from apoptosis and necrosis, since all of them exhibit an elevated population of PI+ cells by flow cytometry analysis, as shown by our results. Necrosis is a passive and uncontrolled cell death pathway customarily induced by extreme environmental stimuli, such as hypoxia. Intercellular contents overflow to the peripheral environment, causing inflammation and tissue damage [39]. Apoptosis is the process by which a cell ceases to grow and divide, and instead enters a process that ultimately results in the controlled death of the cell without spillage of its contents into the surrounding environment [40]. There are two pathways of apoptosis, intrinsic and extrinsic ways, both of which converge, resulting in cell death when the executioner caspase CASP3 is activated [40–42]. Accordingly, we detected the expression of CASP3 in PRMT5-knockdown and negative control MM cell lines, and the results showed no significance, suggesting only an infinitesimal possibility of apoptosis activation. Here, we demonstrate that CASP1 is a possible target of PRMT5 in MM. However, the coenrichment of CASP1 with H4R3me2s remains unknown and needs further research. Similarly, although our results revealed crosstalk between PRMT5 and the CASP1-mediated pyroptosis pathway, the detailed mechanism remains to be determined.

Our results implied the possibility that the PRMT5 inhibitor GSK591 may represent a therapeutic approach for MM. In fact, PRMT5 inhibitors have shown strong therapeutic potential in oncotherapy, with some of them already commercialized. Traditional inhibitors of PRMT5 mainly consist of SAM analogs (DS-437 [43], MTA [44], etc.), CMP5 [45], and CMP5 derivatives (HLCL-61 [19], EPZ01586 [20], etc.). Novel small-molecule PRMT5 inhibitors (GSK591, GSK3326595, etc.) hinder its methyltransferase activity by suppressing the formation of the PRMT5/MEP50 complex. Scientists from the Sanford Burnham Prebys Medical Discovery Institute found that combined treatment with PRMT5 inhibitors and PD-1 inhibitors successfully enhanced the response to antitumor immunity by regulating methylation of interferon-γ (IFN-γ)-inducible protein 16 (IFI16) and its murine homolog IFI204, which are components of cGAS/STING, and by inhibiting transcription of the nucleotide-binding oligomerization domain-like receptor family caspase recruitment domain containing 5 (NLRC5) gene in melanoma [46]. At present, several PRMT5 inhibitors are being examined in clinical trials, including GSK3326595 (phase II clinical), JNJ-64619178, and PF-06939999.

We explored the biological function of PRMT5 in MM and then focused on its mechanism of regulating the pyroptosis pathway both in vitro and in vivo. Taken together, our findings demonstrate that PRMT5 regulates cell pyroptosis by silencing CASP1 in MM, and the PRMT5 inhibitor GSK591 may serve as a potential therapeutic agent for MM.

## Materials and methods

### Cell lines and cell culture

MM cell lines (NCI-H929, RPMI-8226, and U266) were purchased from American Type Culture Collection (ATCC). All cells were cultured in RPMI 1640 medium (Gibco, Carlsbad, CA) supplemented with 10% fetal bovine serum (Gibco) in an atmosphere at 37 °C with 5% CO_2_.

### cDNA preparation and qRT-PCR

Total RNA was extracted using RNA-easy Isolation Reagent (R701-01/02, Vazyme Biotech) and quantified using an ultramicro spectrophotometer (NanoPhotometer-NP80, USA). Reverse transcription was performed using ABScript II RT Mix for qPCR with gDNA Remover (RK20403, Abclonal) according to the manufacturer’s protocol. Quantitative real-time PCR analysis was performed using qPCR SYBR Green Master mix (Q311-02, Vazyme Biotech) with an Applied Biosystems^®^ QuantStudio™ 6 Flex Real-Time PCR System. The relative quantitative value was calculated using the 2^−ΔΔCt^ method. The final result was recorded as fold change. Every sample was run using with three replicates. The primers used for qRT-PCR are described in Supplemental Table 1.

### Patient samples and immunohistochemistry

Bone marrow samples of newly diagnosed MM patients were collected at Nanjing Drum Tower Hospital, Jiangsu Province, China. MM patients with a distinctive pathologic diagnosis, no preoperative systemic or local treatment, and complete follow-up data were selected for inclusion in this study. The paraffin-embedded tissue were deparaffinized, rehydrated, and then subjected to antigen retrieval. The tissue slides were, respectively, incubated with PRMT5 antibodies (1:200; Sigma) and CASP1 antibodies (1:200; CST) overnight at 4 °C. Subsequently, the slides were incubated with horseradish peroxidase (HRP)-conjugated secondary antibody. Each sample was independently scored by two pathologists who were blinded to all the patient clinical data. Based on the percentage of PRMT5- or CASP1-positive tumor cells, the extent of staining was scored 0 (negative), 1 (1–25%), 2 (26–50%), 3 (51–75%), or 4 (76–100%). The final score for each slide was assessed by multiplying the scores for intensity and extent of staining. PRMT5 or CASP1 expression on slides was considered low if the score was <3 and high if the score was >3.

### Transfection of cell lines

PRMT5 shRNA lentivirus was prepared by the co-transfection of 293T cells with pLKO.1 and the packaging plasmids pMD2G and psPAX2. At 48 h after transfection, the viral supernatants were collected. NCI-H929 or U266 cells were incubated with viral supernatants in the presence of polybrene. Positive cells were selected using 1 μg/ml puromycin. The target sequences were PRMT5 shRNA#1: 5ʹ-CCCATCCTCTT CCCTATTAAG-3ʹ and PRMT5 shRNA#2: 5ʹ-GCCCAGTT TGAGATGCCTTAT-3ʹ. A scrambled sequence was used as a negative control.

CASP1 siRNAs were synthesized by SyngenTech Inc. (Beijing, China) and transfected using Lipofectamine 2000 (Invitrogen, USA) according to the manufacturer’s protocol. SiRNA sequences targeting CASP1 were siRNA#1: 5ʹ-GGUGUGGUUUAAAGAUUCATT-3ʹ; siRNA#2: 5ʹ-GAAGACUCAUUGAACAUAUTT-3ʹ; and siRNA#3: 5ʹ-CUCUCAAGGAGUACUUUCUTT-3ʹ.

CASP1 overexpression plasmids and the control plasmid were purchased from SyngenTech Inc. (Beijing, China) and were transfected into cells using Lipofectamine 2000 (Invitrogen, USA) according to the manufacturer’s protocol.

### Protein extraction and western blotting

Cells were washed with PBS and lysed in lysis buffer (20 mM Tris at pH 7.5, 150 mM NaCl, 1% Triton X-100, sodium pyrophosphate, β-glycerophosphate, EDTA, Na_3_VO_4_, leupeptin). The concentration of the protein was quantified using the BCA protein assay (Thermo Fisher Scientific, USA). Western blotting was implemented in accordance with the standard methods [47]. The antibodies used were as follows: PRMT5 (P0493, Sigma), GAPDH (HRP-60004, Proteintech), CASP1 (3866, CST), cleaved-CASP1 (4199, CST), H4R3me2s (ab5823, Abcam), cleaved-CASP3 (9661, CST), N-GSDMD (ab215203, Abcam), IL-1b (66737-1-Ig, Proteintech), and IL-18 (10663-1-AP, Proteintech). Quantifications of all western blotting signals with 3 repeated experiments are shown in Supplemental Fig. 2A–D as histograms.

### GeneChip

According to the manufacturer’s instructions, biotinylated cRNA were prepared according to the standard Affymetrix protocol from 6 μg total RNA (Expression Analysis Technical Manual, 2001, Affymetrix). Following fragmentation, 10 μg of cRNA was hybridized for 16 h at 45 °C on GeneChip Rat Gene 2.0. GeneChip were washed and stained in the Affymetrix Fluidics Station 400. GeneChip were scanned using the Hewlett-Packard GeneArray Scanner G3000 7G. The data were analyzed with RMA using Affymetrix default analysis settings and global scaling as normalization method. The trimmed mean target intensity of each array was arbitrarily set to 100.

### Chromatin immunoprecipitation (ChIP)

Cells were cultured in RPMI-1640 with 10% FBS and then cross-linked with 1% formaldehyde at room temperature. Then, 10–15 min later, the reaction was quenched with glycine for 10 min at a final concentration of 125 mM. Subsequently, the chromatin was sonicated to produce DNA fragments (200–500 bp). For this, 100 μg of chromatin was incubated overnight with rotation at 4 °C with H4R3me2s (Abcam) antibodies or IgG (2 μg) followed by 1 h incubation with protein A Sepharose beads. After washing with low-salt buffer and high-salt buffer, the beads were incubated with elution buffer and proteinase K at 65 °C overnight to immunoprecipitate the DNA. Total DNA fragments were isolated by phenol/chloroform extraction and ethanol precipitation. After isolation, the DNA was diluted in water and subjected to real-time PCR analysis. The primers designed for qRT-PCR are shown in Supplemental Table 2.

### Immunofluorescence

NCI-H929 cells and U266 cells were cultured on glass coverslips, fixed in 4% paraformaldehyde (Sigma) for 30 min and permeabilized using 0.3% Triton X-100 for 30 min at room temperature. After blocking with 5% goat serum in PBS, cells were incubated with PRMT5 antibodies (Sigma) for 60 min and secondary antibodies for 45 min, respectively, in a dark and humid chamber. Then, the cells were washed with PBS, and the nuclei were stained with 4,6,diarnidino-2-phenylindole (DAPI) for 5–10 min. Immunofluorescence images were captured under a fluorescence microscope (BX53, Olympus).

### CCK-8 assay

The indicated cells were seeded into each well at a density of 1000 cells in 96-well plates. We examined cell growth every 24 h using a Cell Counting Kit-8 kit (CCK-8, Dojindo, Japan) after incubation for 1 h at 37 °C. According to the reference wavelength, the absorbance value was detected at 450 nm.

### Flow cytometry

An Annexin V-FITC/PI kit (BD Biosciences) was used to measure apoptosis according to the manufacturer’s protocol. Briefly, 10^5^ cells were collected and washed twice with PBS. A total of 50 µl binding buffer, 5 µl Annexin V-FITC, and 5 µl PI were added to the cell suspension and mixed at room temperature in the dark for 10 min. Apoptosis was measured using a flow cytometer (BD Accuri C6 Plus) within 1 h. The flow cytometry data were analyzed using FlowJo 10.

### Tumor growth in xenografts

In all, 18 female BALB/c nude mice were fed sterilized feed and pure water in a specified pathogen-free environment in an air laminar flow chamber. Feed, bedding, and cages for indoor use were autoclaved and transported through a sterile inlet chamber to exclude any microorganisms. Then, the mice were divided into three groups and subcutaneously inoculated with 1 × 10^7^ NCI-H929 cells transfected with scr shRNA (Group A, *n* = 6), PRMT5 shRNA (Group B, *n* = 6), or PRMT5 shRNA plus CASP1 siRNA (Group C, *n* = 6). All mice were weighed, and xenografts were measured using Vernier calipers every week after injection. Five weeks later, all the mice were euthanized by cervical dislocation, and the subcutaneous tumor xenografts of the mice were collected. The maximum diameter of the tumor xenografts was measured using Vernier calipers.

### Statistical analysis

Statistical analysis was performed using Student’s *t*-test for comparing two groups using GraphPad Prism software. Data are shown as mean ± SD. Differences in the mean values were considered to be significant at *P* < 0.05.

## Supplementary information

Detailed Author Contribution.

Supplemental Figure legends.

Supplemental Fig1.

Supplemental Fig2.

Supplemental Table 1.

Supplemental Table 2.

## Data Availability

All data included in this study are available from the corresponding authors upon request.
