# Correction: PRMT5 regulates cell pyroptosis by silencing CASP1 in multiple myeloma

**DOI:** 10.1038/s41419-021-04373-5

**Published:** 2021-11-22

**Authors:** Tian Xia, Ming Liu, Quan Zhao, Jian Ouyang, Peipei Xu, Bing Chen

**Affiliations:** 1 Department of Hematology, The Affiliated Drum Tower Hospital of Nanjing University Medical School, Nanjing, 210008 Jiangsu People’s Republic of China; 2 The State Key Laboratory of Pharmaceutical Biotechnology, Nanjing University, Nanjing, 210008 People’s Republic of China; 3 Clinical College of Traditional Chinese and Western Medicine, Nanjing University of Chinese Medicine, Nanjing, 210008 Jiangsu People’s Republic of China

**Keywords:** Myeloma, Enzyme mechanisms

Correction to: *Cell Death and Disease* (2021) 12:851; 10.1038/s41419-021-04125-5, published online 16 September 2021

The original version of this article unfortunately contained a mistake. During typesetting the order of corresponding authors was changed. The correct order is Tian Xia, Ming Liu, Quan Zhao, Jian Ouyang, Peipei Xu and Bing Chen. We apologize for the error. The original article has been corrected.

