# Supplementary material for: PRMT5 regulates cell pyroptosis by silencing CASP1 in multiple myeloma
Source: Cell Death Dis. 2021 Sep 16;12(10):851. doi: 10.1038/s41419-021-04125-5 (PMC8445991; doi:10.1038/s41419-021-04125-5)
Supplement: Supplementary file 2 — Supplemental Figure legends. [file 41419_2021_4125_MOESM2_ESM.docx]

**Supplemental Figure legends**

Supplemental Fig.1 Quantifications of cell viability detection by CCK-8 assay. (A) Cell viability analysis of NCI-H929 (a) and U266 (b) cells stably expressing the control or PRMT5 shRNA. (B) Cell viability analysis of NCI-H929 (a) and U266 (b) cells transfected with the control or CASP1-OE plasmid. (C) Cell viability analysis of NCI-H929 (a) and U266 (b) cells transfected with PRMT5 shRNA with or without the co- transfected CASP1 siRNAs. (D) Cell viability analysis of NCI-H929 (a) and U266 (b) cells treated with 0, 1, 5, 10 μM of PRMT5 inhibitor GSK591. Data shown are mean ± SD (n = 3). **P* < 0.05.

Supplemental Fig.2 Quantifications of western blotting signals. (A) Quantitive levels of PRMT5 (a) and cleaved-CASP3 (b) of the indicated groups from Figure 2A&E. (B) Quantitive levels of CASP1 (a) and cleaved-CASP1 (b) of the indicated groups from Figure 3C. (C-D) Quantitive levels of cleaved-CASP1 (a), N-GSDMD (b), IL-1b (c) and IL-18 (d) of the indicated groups from Figure 4C&F. Data shown are mean ± SD (n = 3). **P* < 0.05, ***P* < 0.01, ****P* < 0.001.
